# Characterization and comparison of *Schizosaccharomyces pombe* *cdc15* temperature-sensitive mutants

**DOI:** 10.17912/micropub.biology.001515

**Published:** 2025-03-05

**Authors:** Lesley A. Turner, Aleksandar Vjestica, Alaina H. Willet, Snezhana Oliferenko, Kathleen L. Gould

**Affiliations:** 1 Department of Cell and Developmental Biology, Vanderbilt University School of Medicine, Nashville, TN, US; 2 Temasek Life Sciences Laboratory, Singapore 117604, Singapore; 3 Current address: Center for Integrative Genomics, University of Lausanne, Lausanne, Switzerland; 4 Current address: Randall Centre for Cell and Molecular Biophysics, School of Basic and Medical Biosciences, King’s College London, London, SE1 1UL, UK; 5 Current address: The Francis Crick Institute, 1 Midland Road, London, NW1 1AT, UK

## Abstract

The F-BAR protein Cdc15 is essential for cytokinesis in the fission yeast *Schizosaccharomyces pombe* , playing a key scaffolding role and connecting the actomyosin-based cytokinetic ring to the plasma membrane. Here, we compared *cdc15* temperature-sensitive mutants isolated in multiple genetic screens. We determined the mutations within each *cdc15* mutant allele and analyzed their growth at different temperatures. Additionally, we report a new *cdc15* allele that highlights the requirement for Cdc15 in the recruitment of the early secretory pathway to the cellular division site. The new mutants described here expand the toolkit for studying cytokinesis in *S. pombe* .

**Figure 1. Characterization of mutant alleles.**
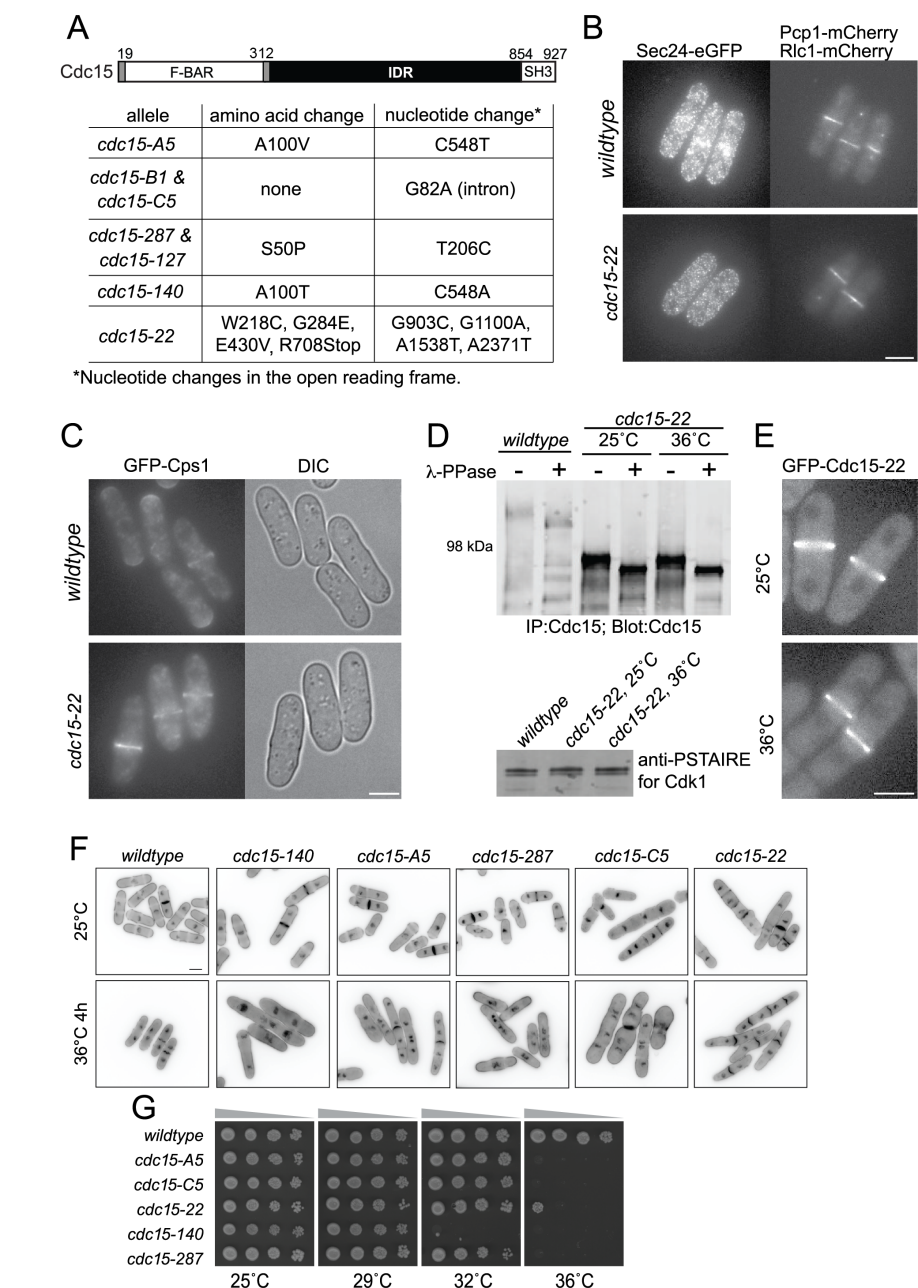
(A) Schematic of gene product (drawn to scale). Numbers indicate amino acid position. Below the schematic is a table of *cdc15* mutations encoded by the indicated temperature-sensitive alleles. (B) Live cell imaging of wildtype and *cdc15-22* cells expressing Sec24-GFP and mCherry-tagged actomyosin ring component Rlc1 and spindle pole body component Pcp1 grown at the permissive temperature of 25°C and shifted to the restrictive temperature of 36˚C for 3 hours. Shown are the maximum projection images of nine 0.5 μm-spaced z-sections imaged at 25˚C or 36˚C. (C) Live cell imaging of wildtype and *cdc15-22* cells expressing GFP-Cps1 grown at the permissive temperature of 25°C and shifted to the restrictive temperature of 36˚C for 3 hours. Shown are the maximum projection images of the z-stacks obtained by epifluorescence imaging at the appropriate temperatures of 25˚C or 36˚C. (D) Immunoprecipitated (IP) Cdc15 proteins treated or not treated with lambda phosphatase (λ-PPase) were separated by SDS-PAGE and immunoblotted ( *top* ). Lysates were blotted for Cdk1 levels as a loading control prior to immunoprecipitations ( *bottom* ). (E) The protein encoded by the *cdc15-22* allele was tagged with GFP at the N-terminus and cells were grown either at 25°C (top) or shifted to 36˚C for 3 hours (bottom) before being imaged live at those temperatures. Shown are the maximum projection images of the nine 0.5 μm spaced z-sections. (F) The indicated strains were grown at 25˚C and shifted to 36˚C for 4 hours. Samples were collected at both temperatures and cells were fixed and stained with DAPI and methyl blue before imaging. (G) The indicated strains were grown in liquid YE at 25°C until they reached mid-log phase and adjusted to the same cell concentrations measured by optical density (Forsburg and Rhind, 2006). Then, 10-fold serial dilutions were made and 2.5 µL of each was spotted on YE agar plates and incubated at the indicated temperatures for 2-5 days prior to imaging. (B, C, E, F) Scale bar, 5 µm.

## Description

In the fission yeast *Schizosaccharomyces pombe, c* ytokinesis requires the function of the plasma membrane (PM)-bound actin- and myosin-based cytokinetic ring (CR) that is assembled at the cell equator at the onset of mitosis. CR constriction at the end of anaphase ultimately results in the physical separation of two daughter cells.

One of the proteins mediating CR attachment to the PM is the essential dimeric F-BAR protein Cdc15 ( Figure 1A, top panel). Cdc15 is one of the first and most abundant components detected at the CR (Fankhauser et al., 1995; Nurse et al., 1976; Wu et al., 2003; Wu and Pollard, 2005). As cells progress into mitosis, the N-terminal dimeric F-BAR domain of Cdc15 oligomerizes, binds membranes, and interacts with the formin Cdc12 and the paxillin-related Pxl1 (Carnahan and Gould, 2003; McDonald et al., 2015; Snider et al., 2022; Snider et al., 2020; Willet et al., 2015). A C-terminal SH3 domain provides a second binding site for Pxl1 and also interacts with other CR components including the C2 domain protein Fic1 (Bhattacharjee et al., 2020; Cortes et al., 2015; Martin-Garcia et al., 2018; Ren et al., 2015; Roberts-Galbraith et al., 2009). Between the F-BAR and SH3 domains lies a predicted intrinsically disordered region (IDR) essential for Cdc15 function (Mangione et al., 2019) . Reducing the amount of Cdc15 , preventing Cdc15 membrane binding and/or F-BAR domain-mediated oligomerization, or deleting the Cdc15 SH3 domain destabilizes the CR during anaphase and leads to cytokinesis failure (Arasada and Pollard, 2011; McDonald et al., 2015; Roberts-Galbraith et al., 2009) . Abrogating Cdc15 function also prevents the enrichment of the early secretory machinery, transitional ER (tER) exit sites, at the nascent division site (Vjestica et al., 2008) .

c *dc15* temperature-sensitive mutants have been isolated in genetic screens for general cell cycle regulators and also in those targeting cytokinesis factors (Balasubramanian et al., 1998; Chang et al., 1996; Nurse et al., 1976) . We isolated a novel *cdc15* allele, *cdc15-22* , in a genetic screen for mutants where the CR assembled and remained stable over an extended period of time but failed to recruit tER marked by the eGFP-tagged COPII coat protein Sec24 ( Figure 1B ). Yet, the cell wall synthase Cps1 / Bgs1 localized normally to the division site in *cdc15-22* cells at the restrictive temperature of 36˚C ( Figure 1C ). The mutations within several *cdc15* mutant alleles, including *cdc15-22* , have not been identified or reported in PomBase (Rutherford et al., 2024) .

To determine the molecular lesions in all *cdc15* temperature-sensitive mutants, we amplified and sequenced the *cdc15* open reading frame (ORF) from each strain ( Figure 1A, bottom panel). In one of the first described alleles (Nurse et al., 1976) , *cdc15-140* , we found a single mutation conferring a single amino acid substitution (A100T) within the F-BAR domain. The same amino acid was changed to a valine in *cdc15-A5* . A distinct mutation within the F-BAR domain was also identified in *cdc15-287* and this was an identical change to that in *cdc15-127* . Since the F-BAR mediates membrane binding, it is likely that the F-BAR domain becomes destabilized at the non-permissive temperature. Two alleles, *cdc15-B1* and *cdc15-C5* , contained the same mutation at the 5'end of an intron. This mutation does not lead to an amino acid substitution. Instead, it likely inhibits splicing and causes a decrease in protein production exacerbated at the non-permissive temperature. Indeed, significantly decreasing Cdc15 levels leads to cytokinesis failure and cell death (Arasada and Pollard, 2011) . The last allele we sequenced was *cdc15-22* . This allele contained three amino acid substitutions and a stop codon at the amino acid 708. Introduction of individual *cdc15-22* mutations into the wild-type *cdc15* locus indicated that the premature stop codon was responsible for the cytokinesis failure of *cdc15-22* cells. We previously found that C-terminal truncations to amino acid 752 or amino acid 710 were viable (Mangione et al., 2019; Roberts-Galbraith et al., 2009) . However, these truncation alleles grew slowly and showed a variety of cell division and morphological defects similar to *cdc15-22* cells.

To validate the sequencing results from *cdc15-22* , we performed an immunoprecipitation and immunoblotting experiment. This confirmed that Cdc15-22 was a shorter protein ( Figure 1D ). However, the overall protein levels were not diminished, in fact they appeared increased, and the truncated protein was still phosphorylated. Cdc15's ability to oligomerize and bind membrane and protein partners is regulated by its phosphorylation state (Bhattacharjee et al., 2023; Bhattacharjee et al., 2020; Fankhauser et al., 1995; Roberts-Galbraith et al., 2010). N-terminal tagging of Cdc15-22 mutant protein with eGFP showed that it was efficiently recruited to the CR suggesting that its phosphoregulation was largely intact ( Figure 1E ).

To compare cellular phenotypes of the strains, we examined each mutant by staining for nuclei and septa at the permissive temperature of 25°C or after the shift to the restrictive temperature of 36˚C for 4 hours. The phenotypes of the *cdc15-140* , *cdc15-A5* , and *cdc15-287* cells were comparable to each other, with an accumulation of multiple nuclei and occasionally a septa at the restrictive temperature ( Figure 1F ). In contrast, the *cdc15-C5* and *cdc15-22* cultures contained cells with multiple nuclei and septa even at 25˚C and cells that became wider and bulged at 36˚C ( Figure 1F ).

We next determined the range of temperature-sensitivity of each *cdc15* allele. All *cdc15* alleles grew less well than wildtype cells at 36°C, with *cdc15-140* showing the greatest temperature-sensitivity ( Figure 1G ).

In sum, we have provided further information on range of *cdc15* mutants as well as initial characterization of previously uncharacterized *cdc15* mutants that expand the repertoire of reagents which can be used to study cytokinesis and subcellular polarization of the early secretory pathway compartments in *S. pombe* .

## Methods

*S. pombe* methods

*S. pombe* strains were grown in yeast extract (YE) and standard *S. pombe* mating, sporulation, and tetrad dissection techniques were used to construct new strains (Forsburg and Rhind, 2006) . Spot assays were performed twice with reproducible results.

The marker reconstitution mutagenesis strategy described in (Tang et al., 2011) was used to isolate *cdc15-22* . Briefly, a non-functional fragment of the selective marker *his5* (containing the promoter and the N-terminal ORF fragment), together with the entire *ura4* gene were inserted at the 3' end of the native *cdc15* genomic locus, in the *his5-* *ura4 ^-^* mutant genetic background. This strain was obtained by transforming the HpaI-linearized plasmid cdc15-pH5-C∆ and we denoted the locus as *cdc15-his5C∆::ura4 ^+^* . In parallel, we performed error-prone PCR of the *cdc15* genomic sequence fused to the 3′-UTR and the C-terminal part of *his5 ^+^* from the plasmid cdc15-pH5-C+, at high Mg ^2+^ concentration. The mutagenized amplicon was transformed into *cdc15-his5C∆::ura4 ^+^* locus and the homologous recombination within *cdc15-* and *his5* -encoding sequences resulted in the replacement of the genomic *cdc15* sequence with a library of PCR fragments. Such recombination events were selected based on the restoration of histidine prototrophy. We screened all resulting colonies for temperature-sensitive *cdc15* alleles showing impaired recruitment of the tER to the cellular division site.

To tag the mutant Cdc15-22 protein with eGFP we used the 3'region *^cdc15^* -5'region *^cdc15^* -eGFP- *cdc15* ^ORF^ -pJK210 plasmid that upon SnaBI linearization has homology arms to the 5'UTR and the 3'UTR of *cdc15* gene, introducing the *eGFP- cdc15* construct and the *ura4+* cassette at the *cdc15* locus.

Molecular biology methods

*cdc15* alleles were amplified using an oligonucleotide 123 bp upstream of the start site (GATAGGCAACGGTTGCTAGG) and 142 bp downstream of the stop codon (ACGAAGCTTAGACCATGACG) (Integrated DNA technologies). The PCR products were each sequenced by Plasmidsaurus (Eugene, OR) using Oxford Nanopore Technology with custom analysis and annotation.

The *cdc15-pH5-C∆* plasmid was obtained through overlap-extension PCR using primers prSO1372(ccgctcgagACACTGATTACCCTCTTTTCTAG), prSO1373(ccactataaccaccattgttAACATTGTTAATAAAATAAAATAAAATATATTCC), prSO1374(tttattttattaacaatgttAACAATGGTGGTTATAGTGGATCG), prSO1375(CGGAATTCctaTACCGTCTGAACAAAGTTCG) and the amplicon was introduced into the pH5-C∆ plasmid (Tang et al., 2011) using XhoI and EcoRI restriction sites. The plasmid cdc15-pH5-C+ was obtained using prSO1376(cgcggatccGGATCGAACATTAAATTTAACG) and prSO1377(gctacggatatcCTATACCGTCTGAACAAAGTTCG) to amplify *cdc15* locus and cloned into pH5-C+ backbone *(Ta* ng et al., 2011) using BamHI and EcoRV restriction sites.

For the N-terminal eGFP tagging of Cdc15-22 protein variant, we amplified the mutant allele ORF sequence with prSO712 (CGCGGATCCATGGAGGTTAATGGAGTCTCTCAATCTG) and prSO2584 (tccccgcggCTATACCGTCTGAACAAAGTTCGACGGG) and used to clone it downstream of eGFP in pJK210 vector using BamHI and SacII restriction sites. We amplified the 3'region of *cdc15* using prSO2585 (ccatcgatACACTGATTACCCTCTTTTCTAGATG) and prSO2586 (tcgcccgggttgttctgcggagtacGTACCTTGATTTTAATTTACTAAAATCGG) and 5' region of *cdc15* using prSO2587 (ctccgcagaactacGTATCTGCTAGCAAGTTTGAATGAAAATAGG) and prSO2588 (tcgcccgggAACCTCCATTTTGTTATTGAAAGAAC) and cloned them in front of the eGFP using ClaI, SnaBI and XmaI restriction sites to obtain the final plasmid 3'region *^cdc15^* -5'region *^cdc15^* -eGFP- *cdc15* ^ORF^ -pJK210 which upon linearization with SnaBI targeted the native cdc15 locus.

Microscopy and image analysis

Strains for fixed-cell imaging experiments were grown at 25°C in YE and then shifted to 36°C for 4 hours. Cells were fixed with 70% ethanol for DAPI and methyl blue (MB) staining as described previously (Roberts-Galbraith et al., 2009). Images were acquired using a Zeiss Axio Observer inverted epifluorescence microscope with Zeiss 63× oil objective (1.46 NA) and captured using Zeiss ZEN 3.0 (Blue edition) software. A singular medial Z slice was obtained. All images were further processed using ImageJ (Schindelin et al., 2012) . All imaging experiments were repeated twice.

Live imaging of *cdc15-22* strains was performed on epifluorescence microscopy setup using mercury lamp as an illumination source with appropriate sets of filters on a Zeiss Axiovert 200M microscope (Carl Zeiss, Inc.) using a Plan Apochromat 100X, 1.4 N.A. objective lens, CoolSnap camera (Photometrics, Tucson, AZ) and Uniblitz shutter driver (Photonics, Rochester, NY) under the control of Metamorph software package (Universal Imaging, Sunnyvale, CA). When required, imaging was performed at 36˚C using an objective heater system (Bioptechs, Butler, PA, USA). We acquired whole cell image stacks that consisted of 9 z-sections with 0.5 µm spacing for red and green fluorophores. The z-stack maximum or average projection images were obtained by ImageJ 1.46b software package (http://rsb.info.nih.gov/ij/; National Institutes of Health, Bethesda, MD, USA).

Protein methods

Cell pellets (15 OD) were snap-frozen in dry ice–ethanol baths. Pellets were resuspended in 1 ml and lysates were prepared by bead disruption (Fastprep cell homogenizer; MP Biomedicals) in NP-40 buffer (6 mM Na _2_ HPO _4_ , 4 mM NaH _2_ PO _4_ , 1% NP-40, 150 mM NaCl, 2 mM EDTA, 50 mM NaF, 4 mg/mL leupeptin, 0.1 mM Na _3_ VO _4_ ) with the addition of 1 mM PMSF (Sigma-Alrdrich; P7626), 2 mM benzamidine (Sigma-Alrdrich; B6506), and 0.5 mM diisopropyl fluorophosphate (Sigma-Aldrich; D0879-1G). Immunoprecipitations and lambda phosphatase collapse were performed as described (Bhattacharjee et al., 2023) . Briefly, lysates were incubated with anti- Cdc15 serum (Roberts-Galbraith et al., 2009) for 2 h at 4°C and then protein A sepharose beads (GE Healthcare; 17-5280-04) for 30 minutes. Beads were washed three times with NP-40 buffer and two times with phosphatase buffer (150 mM NaCl, 50 mM Hepes pH 7.4) before being split in two and added to either lambda phosphatase reaction or control. Lambda phosphatase collapse was performed according to manufacturer's protocol (New England Biolabs; P0753). Reactions were stopped by the addition of gel sample buffer.

Protein samples were resolved by SDS-PAGE and transferred to polyvinylidene fluoride (PVDF) membrane (Immobilon P, EMD Millipore). Anti- Cdc2 (PSTAIRE) mouse monoclonal antibody at 1:10,000 dilution (Sigma-Aldrich; P7962) or anti- Cdc15 (1-405) rabbit polyclonal antibody at 1:10,000 dilution (Roberts-Galbraith et al., 2009) were used as primary antibodies in immunoblotting. Secondary antibodies were conjugated to IRDye800 or IRDye680 (LI-COR Biosciences). Blotted proteins were detected via an Odyssey CLx instrument (LI-COR Biosciences).

## Reagents

The strains used in this study and their genotypes are listed below.

**Table:** 

**Strain**	**Genotype**	**Source**
KGY114-2	* cdc15-C5 ura4-D18 h ^+^*	This study
KGY188	* cdc15-140 h ^-^*	Nurse et al., 1976
KGY282-2	* cdc15-A5 ura4-D18 h ^90^*	This study
KGY642-3	* cdc15-127 h ^-^*	Nurse et al., 1976
KGY749	* cdc15-287 h ^-^*	Chang et al., 1996
KGY1077	* cdc15-B1 ura1 leu1-32 mam2::LEU2 ade6-M216 h ^90^*	Balasubramanian et al., 1998
KGY1078	* cdc15-C5 ura1 leu1-32 mam2::LEU2 ade6-M216 h ^90^*	Balasubramanian et al., 1998
KGY1079	* cdc15-A5 ura1 leu1-32 mam2::LEU2 ade6-M216 h ^90^*	Balasubramanian et al., 1998
SO6279	* cdc15-22:ura4 ^+^ :his5 ^+^ ade? his5? leu1-32 ura4-D18* *h ^+^*	This study
SO5986	* sec24-GFP:ura4+ cdc15 :his5C∆:ura4+ his5- h? *	This study
SO7141	*cdc15-22:his5+:ura4+ sec24-GFP rlc1-mCherry:ura4+ pcp1-mCherry:ura4+ h? *	This study
SO7350	*eGFP-cdc15-22:ura4+ h? *	This study
SO628 *5*	* cdc15-22:his5+:ura4+ GFP- cps1 :kanR h? *	This study
